# Bacteriophage Interactions With Epithelial Cells: Therapeutic Implications

**DOI:** 10.3389/fmicb.2020.631161

**Published:** 2021-01-18

**Authors:** Andrzej Górski, Jan Borysowski, Ryszard Miȩdzybrodzki

**Affiliations:** ^1^Bacteriophage Laboratory, Hirszfeld Institute of Immunology and Experimental Therapy, Polish Academy of Sciences (HIIET PAS), Wrocław, Poland; ^2^Phage Therapy Unit, Hirszfeld Institute of Immunology and Experimental Therapy, Polish Academy of Sciences (HIIET PAS), Wrocław, Poland; ^3^Infant Jesus Hospital, The Medical University of Warsaw, Warsaw, Poland; ^4^Department of Clinical Immunology, Transplantation Institute, Medical University of Warsaw, Warsaw, Poland

**Keywords:** phage therapy, epithelial cells, inflammation, eukaryotic cells, cytokines

## Introduction

Since their discovery, phages have been considered to be predators of bacteria and unable to infect eukaryotic cells. However, in the 1970s, Merril already suggested that phages may cause metabolic alterations in mammalian cells (Merril, 1973). This report paved the way for further studies showing that indeed phages may have affinity to eukaryotic cells.

## Phages Interact With the Cells of the Immune System

In 2005 we formulated a hypothesis pointing to the immunomodulatory activity of intestinal phages (mostly coliphages) and their ability to migrate from the gut and exert immunomodulating action on various organs and tissues through the effect of phage proteins on eukaryotic cells (Górski and Weber-Dabrowska, 2005). In the years since then, evidence has been accumulating shedding more light on those phage interactions demonstrating phage ability to downregulate reactive oxygen species production by phagocytes, alterations of expression of surface markers, inhibition of migration of immune cells to antigen sites and immunomodulation of cytokine production. Phages do not replicate in eukaryotic cells so those effects must be mediated by their outer proteins. Barr (2017) have confirmed that phages can bypass epithelial cell layers, disseminate, and manipulate our immune system (Barr, 2017). *In vitro* studies of Van Belleghem et al. (2017) have demonstrated diversity of phage action on the immune system. Recently, using the zebrafish model Cafora et al. (2020) showed anti-inflammatory effects of phages independent of their well-known anti-bacterial action. The interactions of phages with the cells of the immune system have been summarized (Górski et al., 2017; Van Belleghem et al., 2018).

## Phages May Bind to and Penetrate Epithelial and Other Cell Types

Data have also been slowly accumulating demonstrating phage interactions with other cell types. Our group has provided evidence that phages may bind to melanoma cells causing inhibition of experimental lung metastases in mice. This phenomenon probably depends on specific interactions between the Lys-Gly-Asp (KGD) motif of the T4 phage protein 24 and beta3 – integrin receptors on target cells (Dabrowska et al., 2004). Furthermore, the T4 phage and its mutant HAP1 cause inhibition of mouse and human melanoma cell migration on fibronectin and matrigel (Dabrowska et al., 2009). Interestingly, the anti-bacterial activity of phages may significantly be enhanced in the presence of human cells (Shan et al., 2018).

In the past decade it has been established that phages (T4 as well as T3, T5, T7, SPO1, and P22 phages) not only bind to but also penetrate eukaryotic cells including epithelial cells from the gut, lung, liver, brain, and kidney (Nguyen et al., 2017). The mechanisms of phage uptake and their intracellular migration pathways have recently been discussed (Zaczek et al., 2020).

Bichet et al. (2020) examined the rate of T4 phage uptake by different cell types. A549 lung epithelial cells showed the highest accumulation of phages, while endothelial cell types showed intermediate levels. HT-29 and BJ cells representing epithelial and fibroblast cells showed little or no accumulation. Furthermore, smaller sized phages showed an increased rate of uptake. In contrast to Shan et al., the authors noted that adsorption with *in vitro* cell layers caused inactivation of a high proportion of phages. The authors thus conclude that “phage accumulation by those cell layers represents a major sink for circulating phages in the body.” On the other hand, recent data from Oie et al. (2020) emphasize the significance of liver sinusoidal endothelial cells in the uptake of phages.

## Phages May Induce Production of Cytokines and Other Biologically Active Factors in Epithelial Cells

The data indicating that phages may cross epithelial cell barriers and penetrate the cells raises the question of any consequences of this phenomena for the functions of cells in which phages migrate and accumulate. Available data suggest that phage penetration does not cause cytotoxicity, although one report suggested that phages may cause some inhibition of epithelial cell proliferation. At the same time it is becoming clear that phages may stimulate production of cytokines and other biologically active factors in epithelial cells. The HT-29 colon cell line stimulated with *E. coli* phage may produce Il-8 and Migration Inhibitory Factor (MIF), while under the same condition blood mononuclear cells (MNC) release IL-6, IL-10, and TNF-alpha (Khan Mirzaei et al., 2016).

Pincus et al. (2015) showed that a staphylococcal phage induces interferon gamma in primary human keratinocyte cultures but not in blood MNC. Likewise, *E. coli* phages elicited marked production of IFN gamma and IL-12 by lung cells but not MNC (Dufour et al., 2019).

Phages have also been shown to abolish inflammatory responses induced in epithelial cells. A staphylococcal phage suppressed LPS-dependent inflammatory cytokine production by bovine mammary epithelial cells in culture while phages alone induced only low expression of cytokines. Phage-mediated reaction was associated with downregulation of NF-kappaB signaling (phages suppressed LPS-induced phosphorylation of NF-kappaB p 65), thus confirming our earlier data (Górski et al., 2017; Zhang et al., 2018).

Trend et al. (2017) studied the effects of a *Pseudomonas* phage on cytokine synthesis by human airway epithelial cells. No significant inflammatory cytokine production could be detected (IL-1, IL-6, IL-8). Interestingly, the phage decreased the rate of cell apoptosis in culture.

Our group has studied the effect of T4 and A5/80 (staphylococcal) phages on the expression of immunologically relevant genes by CaCo-2 cells in culture. The most remarkable effect was a marked increase in the expression of the *DEFB4A* gene caused by the T4 but not the A5/80 phage (Borysowski et al., 2020). This gene codes for human beta-defensin 2 (hBD2), a potent antimicrobial peptide synthesized by epithelial cells that exhibits a wide range of activities against viruses, bacteria and fungi. Recently, hBD2 has been shown to suppress key features of asthma in a murine model of allergic airways disease (Pinkerton et al., 2020). In addition, it mediates protection against *Mycobacterium avium* in a mouse lung infection model (Shiozawa et al., 2020), promotes wound healing (Shelley et al., 2020) and inhibits biofilm production (Parducho et al., 2020). Furthermore, Koeninger et al. (2020) demonstrated that hBD2 may be applied as a well-tolerated systematically administered anti-inflammatory agent in the treatment of experimental colitis in mice. Earlier we found that, in A549 cells, both phages induce overexpression of the *HSPA1* gene encoding heat shock protein HSp72, a cellular chaperone upregulated in response to cellular stress having cytoprotective functions. Moreover, the A5/80 phage induced *TLR10* gene expression while the T4 phage upregulated the expression of *TLR2* and *IL-10* genes (Borysowski et al., 2019).

It is well-known that small doses of molecules associated to pathogens may elicit immune response (e.g., endotoxin). Therefore, phage purity is essential to avoid false results.

## Practical Implications of Phage Interactions With Epithelial Cells

Barr et al. (2013) have presented the phage adherence to mucus model that provides non-host bacterial immunity on mucosal surfaces. This model encompasses a symbiotic relationship between phage and mammalian host providing antimicrobial defense protecting mucosal surfaces. Studies on those interactions are at an early stage and data accrued so far need extension and confirmation. Nevertheless, this area of research appears to be promising and offers perspectives for practical implementation in the treatment of human disease. This assumption is confirmed by the data showing that phage interactions with epithelial cells do not cause cell injury while anti-inflammatory effects may prevail. Thus, phages could be used to control epithelial inflammation which is a common phenomenon in a variety of human disorders leading to chronic systemic inflammation occurring in autoimmune diseases, as well as bacterial, viral, and fungal infections (Uluçkan and Wagner, 2017). Of particular interest are preliminary data which suggest that phages may stimulate epithelial cells to produce biologically active mediators like hBD2. Recently, this protein has been the subject of intense studies, the results of which suggest that it could be applied as an antimicrobial agent and as an immunomodulator in asthma and autoimmune disorders.

It is known that lung alveolar epithelial cells are critical in protection of the respiratory tract against pathogens (Chuquimia et al., 2013). The coronavirus propagates within those cells inducing their apoptosis and death (Mason, 2020) As mentioned above, phages are capable of entering those cells and may inhibit their apoptosis (Trend et al., 2017; Bichet et al., 2020). Therefore, one could hypothesize that phages may protect lung alveolar cells against coronavirus-induced injury.

Furthermore, it should be kept in mind that phages may mediate phage-specific action on mammalian cells so their effect on those cells may vary between different phages and may also be cell and tissue – specific (Górski et al., 2019).

## Discussion

It is becoming apparent that phage interactions with epithelial cells may be relevant for maintaining immune homeostasis, as suggested earlier (Górski and Weber-Dabrowska, 2005). Therefore, disturbances of the composition and functions of the body phageome may eventually lead to disease. Furthermore, it may be envisaged that biologically active phages targeting epithelial cells could be used in the treatment of disorders where epithelial cell layers become dysfunctional due to infammatory processes. More in-depth studies are needed to confirm and extend these assumptions.

## Author Contributions

AG: conceptualization and draft of the manuscript. JB and RM: contributions to original draft. All authors: contributed to the article and approved the submitted version.

## Conflict of Interest

AG, JB, and RM are co-inventors of patents owned by the Hirszfeld Institute of Immunology and Experimental Therapy.

## References

[B1] Barr J. J. (2017). A bacteriophages journey through the human body. Immunol. Rev. 279, 106–122. 10.1111/imr.12565 28856733

[B2] Barr J. J. Auro R. Furlan M. Whiteson K. L. Erb M. L. Pogliano J.. (2013). Bacteriophage adhering to mucus provide a non-host-derived immunity. Proc. Natl. Acad. Sci. U.S.A. 110, 10771–10776. 10.1073/pnas.1305923110 23690590 PMC3696810

[B3] Bichet M. C. Chin W. H. Richards W. Lin Y. W. Avellaneda-Franco L. Hernandez C. A. (2020). Bacteriophage uptake by Eukaryotic cell layers represents a major sink for phages during therapy. bioRxiv. Available online at: https://www.biorxiv.org/content/10.1101/2020.09.07.286716v1 (accessed December 10, 2020).

[B4] Borysowski J. Miedzybrodzki R. Przybylski M. Owczarek B. Weber-Dabrowska B. Gorski A. (2020). The effects of T4 and A5/80 phages on the expression of immunologically important genes in differentiated Caco-2 cells. Adv. Hyg. Exp. Med. 74, 371–376, 10.5604/01.3001.0014.3919

[B5] Borysowski J. Przybylski M. Miedzybrodzki R. Owczarek B. Górski A. (2019). The effects of bacteriophages on the expression of genes involved in antimicrobial immunity. Adv. Hyg. Exp. Med. 73, 414–420. 10.5604/01.3001.0013.4081

[B6] Cafora M. Brix A. Forti F. Loberto N. Aureli M. Briani F.. (2020). Phages as immunomodulators and their promising use as anti-inflammatory agents in a cftr loss-of-function zebrafish model. J. Cyst. Fibros. 10.1016/j.jcf.2020.11.017. [Epub ahead of print]. 33298374

[B7] Chuquimia O. D. Petursdottir D. H. Periolo N. Fernández C. (2013). Alveolar epithelial cells are critical in protection of the respiratory tract by secretion of factors able to modulate the activity of pulmonary macrophages and directly control bacterial growth. Infect. Immun. 81, 381–389. 10.1128/IAI.00950-12 23147039 PMC3536158

[B8] Dabrowska K. Opolski A. Wietrzyk J. Switala-Jelen K. Boratynski J. Nasulewicz A.. (2004). Antitumor activity of bacteriophages in murine experimental cancer models caused possibly by inhibition of beta3 integrin signaling pathway. Acta Virol. 48, 241–248. 15745047

[B9] Dabrowska K. Skaradziński G. Jończyk P. Kurzepa A. Wietrzyk J. Owczarek B.. (2009). The effect of bacteriophages T4 and HAP1 on *in vitro* melanoma migration. BMC Microbiol. 9:13. 10.1186/1471-2180-9-13 19154575 PMC2639589

[B10] Dufour N. Delattre R. Chevallereau A. Ricard J. D. Debarbieux L. (2019). Phage therapy of pneumonia is not associated with an overstimulation of the inflammatory response compared to antibiotic treatment in mice. Antimicrob. Agents Chemother. 63, e00379–e00319. 10.1128/AAC.00379-19 31182526 PMC6658787

[B11] Górski A. Dabrowska K. Miedzybrodzki R. Weber-Dabrowska B. Lusiak-Szelachowska M. Jonczyk-Matysiak E. Borysowski J. (2017). Phages and immunomodulation. Future Microbiol. 12, 905–914. 10.2217/fmb-2017-0049 28434234

[B12] Górski A. Miedzybrodzki R. Jonczyk-Matysiak E. Zaczek M. Borysowski J. (2019). Phage-specific diverse effects of bacterial viruses on the immune system. Future Microbiol. 14, 1171–1174. 10.2217/fmb-2019-0222 31535921 PMC6802706

[B13] Górski A. Weber-Dabrowska B. (2005). The potential role of endogenous bacteriophages in controlling invading pathogens. Cell. Mol. Life Sci. 62, 511–519. 10.1007/s00018-004-4403-6 15747058 PMC11365883

[B14] Khan Mirzaei M. Haileselassie Y. Navis M. Cooper C. Sverremark-Ekström E. Nilsson A. S. (2016). Morphologically distinct *Escherichia coli* bacteriophages differ in their efficacy and ability to stimulate cytokine release *in vitro*. Front. Microbiol. 7:437 10.3389/fmicb.2016.01974 27065990 PMC4814447

[B15] Koeninger L. Armbruster N. S. Brinch K. S. Kjaerulf S. Andersen B. Langnau C.. (2020). Human β-Defensin 2 mediated immune modulation as treatment for experimental colitis. Front. Immunol. 11:93. 10.3389/fimmu.2020.00093 32076420 PMC7006816

[B16] Mason R. J. (2020). Pathogenesis of COVID-19 from a cell biology perspective. Eur. Respir. J. 55:2000607. 10.1183/13993003.00607-2020 32269085 PMC7144260

[B17] Merril C. R. (1973). Effects of bacteriophage on eukaryotes. Johns Hopkins Med. J. Suppl. 2, 73–80. 4802456

[B18] Nguyen S. Baker K. Padman B. S. Patwa R. Dunstan R. A. Weston T. A.. (2017). Bacteriophage transcytosis provides a mechanism to cross epithelial cell layers. mBio. 8, e01874–e01817. 10.1128/mBio.01874-17 29162715 PMC5698557

[B19] Oie C. I. Wolfson D. L. Yasunori T. Dumitriu G. Sorensen K. K. McCourt P. A.. (2020). Liver sinusoidal endothelial cells contribute to the uptake and degradation of entero bacterial viruses. Sci. Rep. 10:898. 10.1038/s41598-020-57652-0 31965000 PMC6972739

[B20] Parducho K. R. Beadell B. Ybarra T. K. Bush M. Escalera E. Trejos A. T.. (2020). The antimicrobial peptide human Beta-Defensin 2 inhibits biofilm production of *Pseudomonas aeruginosa* without compromising metabolic activity. Front. Immunol. 11:805. 10.3389/fimmu.2020.00805 32457749 PMC7225314

[B21] Pincus N. B. Reckhow J. D. Saleem D. Jammeh M. L. Datta S. K. Myles I. A. (2015). Strain specific phage treatment for *Staphylococcus aureus* infection is influenced by host immunity and site of infection. PLoS ONE 10:e0124280. 10.1371/journal.pone.0124280 25909449 PMC4409319

[B22] Pinkerton J. W. Kim R. Y. Koeninger L. Armbruster N. S. Hansbro N. G. Brown A. C.. (2020). Human β-defensin-2 suppresses key features of asthma in murine models of allergic airways disease. Clin. Exp. Allergy. 10.1111/cea.13766. [Epub ahead of print]. 33098152

[B23] Shan J. Ramachandran A. Thanki A. M. Vukusic F. Barylski J. Clokie M. (2018). Bacteriophages are more virulent to bacteria with human cells than they are in bacterial culture; insights from HT-29 cells. Sci. Rep. 8:5091. 10.1038/s41598-018-23418-y 29572482 PMC5865146

[B24] Shelley J. R. Davidson D. J. Dorin J. R. (2020). The dichotomous responses driven by β-Defensins. Front. Immunol. 11:1176. 10.3389/fimmu.2020.01176 32595643 PMC7304343

[B25] Shiozawa A. Kajiwara C. Ishii Y. Tateda K. (2020). N-acetyl-cysteine mediates protection against *Mycobacterium avium* through induction of human β-defensin-2 in a mouse lung infection model. Microbes Infect. 22, 567–575. 10.1016/j.micinf.2020.08.003 32882411

[B26] Trend S. Fonceca A. M. Ditcham W. G. Kicic A. Cf A. (2017). The potential of phage therapy in cystic fibrosis: Essential human-bacterial-phage interactions and delivery considerations for use in *Pseudomonas aeruginosa*-infected airways. J. Cyst. Fibros. 16, 663–670. 10.1016/j.jcf.2017.06.012 28720345

[B27] Uluçkan Ö. Wagner E. F. (2017). Chronic systemic inflammation originating from epithelial tissues. FEBS J. 284, 505–516. 10.1111/febs.13904 27650997

[B28] Van Belleghem J. D. Clement F. Merabishvili M. Lavigne R. Vaneechoutte M. (2017). Pro- and anti-inflammatory responses of peripheral blood mononuclear cells induced by *Staphylococcus aureus* and *Pseudomonas aeruginosa* phages. Sci. Rep. 7:8004. 10.1038/s41598-017-08336-9 28808331 PMC5556114

[B29] Van Belleghem J. D. Dabrowska K. Vaneechoutte M. Barr J. J. Bollyky P. L. (2018). Interactions between bacteriophage, bacteria, and the mammalian immune system. Viruses 11:10. 10.3390/v11010010 30585199 PMC6356784

[B30] Zaczek M. Górski A. Szkaradzinska A. Lusiak-Szelachowska M. Weber-Dabrowska B. (2020). Phage penetration of eukaryotic cells: practical implications. Future Virol. 11, 745–760; 6. 10.2217/fvl-2019-0110

[B31] Zhang L. Hou X. Sun L. He T. Wei R. Pang M. (2018). Staphylococcus aureus bacteriophage suppresses LPS-induced inflammation in MAC-T bovine mammary epithelial cells. Front. Microbiol. 9:1614 10.3389/fmicb.2018.01614 30083140 PMC6064726

